# The elusive brain perivascular fibroblast: a potential role in vascular stability and homeostasis

**DOI:** 10.3389/fcvm.2023.1283434

**Published:** 2023-11-24

**Authors:** Maria J. Sosa, Andy Y. Shih, Stephanie K. Bonney

**Affiliations:** ^1^ Center for Developmental Biology and Regenerative Medicine, Seattle Children’s Research Institute, Seattle, WA, United States; ^2^ Department of Pediatrics, University of Washington, Seattle, WA, United States; ^3^ Department of Bioengineering, University of Washington, Seattle, WA, United States

**Keywords:** perivascular fibroblasts, perivascular space, perivascular macrophages, mural cells, astrocytes, cerebral blood flow, vascular basement membrane, perivascular waste clearance

## Abstract

In the brain, perivascular fibroblasts (PVFs) reside within the perivascular spaces (PVSs) of arterioles and large venules, however their physiological and pathophysiological roles remain largely unknown. PVFs express numerous extracellular matrix proteins that are found in the basement membrane and PVS surrounding large diameter vessels. PVFs are sandwiched between the mural cell layer and astrocytic endfeet, where they are poised to interact with mural cells, perivascular macrophages, and astrocytes. We draw connections between the more well-studied PVF pro-fibrotic response in ischemic injury and the less understood thickening of the vascular wall and enlargement of the PVS described in dementia and neurodegenerative diseases. We postulate that PVFs may be responsible for stability and homeostasis of the brain vasculature, and may also contribute to changes within the PVS during disease.

## Introduction

Perivascular fibroblasts (PVFs) surround the wall of pial arteries and veins on the brain surface, and extend onto parenchymal arterioles and large diameter venules as they penetrate into the central nervous system (1–3). They reside within the perivascular space (PVS) (also called the Virchow-Robin space in superficial layers of the cortex) which is an extracellular matrix (ECM) and cerebral spinal fluid (CSF) filled space encircling arterioles and venules (4). Although PVFs are found within the PVS, their role in this location, and how they support vessel function in health and contribute to vascular pathology in disease remains poorly understood. Here we will discuss how PVFs may maintain vessel integrity, highlighting new data on the organizational and morphological characteristics of PVFs, their transcriptional profiles, and parallels in development and disease.

## Morphology and vascular organization of perivascular fibroblasts

“Flattened adventitial cells” were first described to surround penetrating vessels in the brain in 1969 and were later characterized as pial fibroblasts that were continuous with the overlying meninges, forming a sheath around arteries and veins (5–7). This pial sheath was initially described to dive mainly along arterioles into the brain forming a continuous connection with intracerebral arterioles and arteries in the subarachnoid space. More recent studies using fibroblast reporter mouse lines (Col1a1-GFP and Col1a2-CreER) described PVFs as having flattened somata and lamella that create a sheath around arterioles and venules in the central nervous system (CNS) (3). Further, we now know that PVFs are likely derived from pial fibroblasts during development (8). Collectively these studies have confirmed the identity of these “flattened adventitial cells” on penetrating vessels as being PVFs, likely derived from the overlying pia.

**Figure 1:**
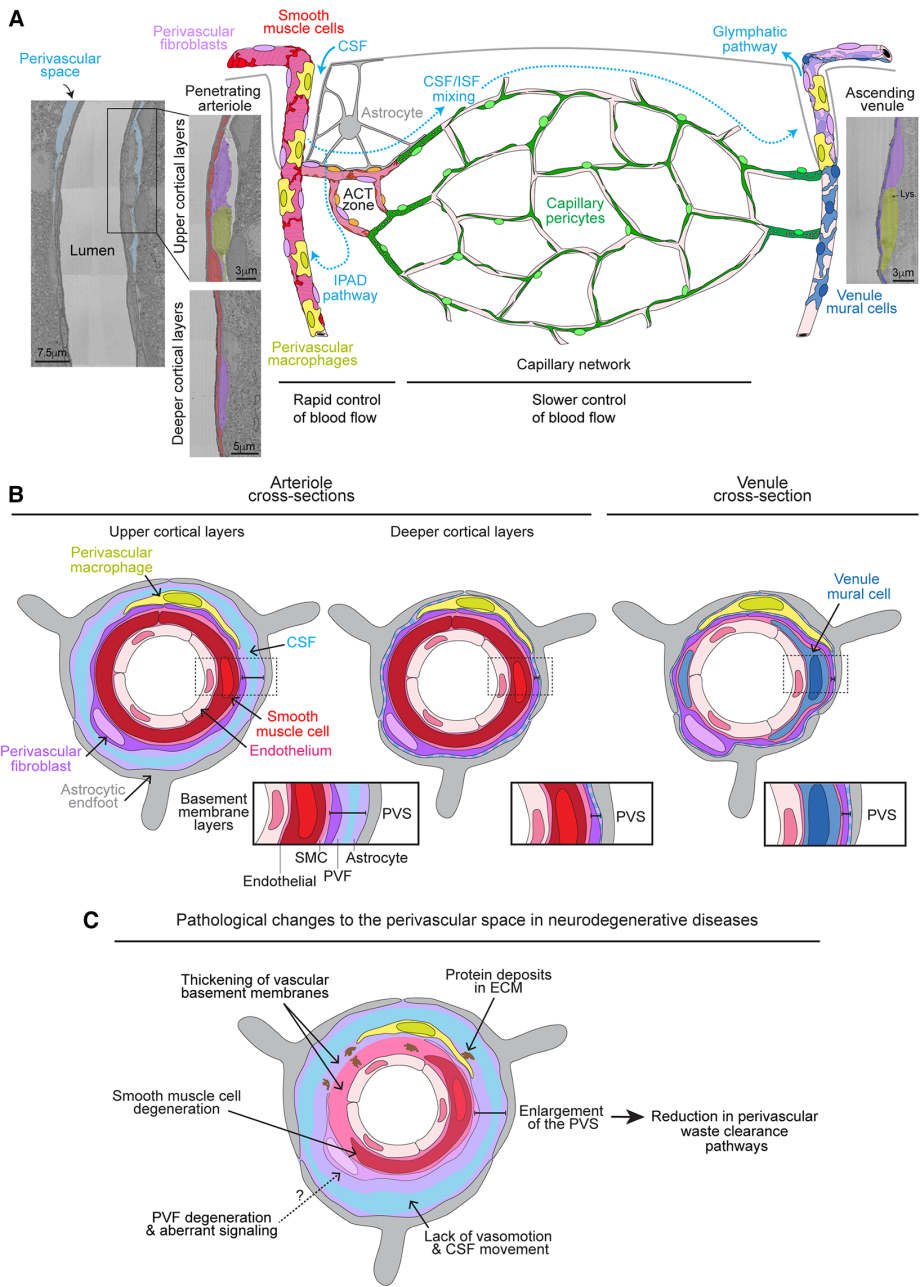
Organization of perivascular fibroblasts on the brain vasculature and role in perivascular space pathology. (**A**) Graphic depicting the vascular organization of perivascular fibroblasts (PVFs) on the brain vasculature accompanied with respective electron micrographs of a penetrating arteriole and ascending venule from the MICrONS 3D electron microscopy (EM) dataset (9, 10) (Arteriole coordinates: 174937, 111854, 19349; Venule coordinates: 296137, 112937, 20593). PVFs (purple) lie within the perivascular space (PVS) and surround smooth muscle cells (red) on penetrating arterioles, ensheathing pericytes (orange) on arteriole capillary transition (ACT) zone, and venule mural cells (blue) on ascending venules. PVFs are not found on capillary vessels where capillary pericytes (green) reside. The cerebral spinal fluid (CSF; light blue)-filled PVS is apparent around arterioles in the upper layers of the cortex and becomes less obvious in deeper cortical layers. Vasomotor oscillations of arteries and arterioles draw CSF into the PVS and brain parenchyma where it mixes with interstitial fluid (ISF) and waste, ultimately exiting out of the brain via the glymphatics or intramural periarterial drainage (IPAD) pathways. Perivascular macrophages (PVMs) (yellow) can be distinguished by the presence of lysosomes (Lys) (11) and also reside within the perivascular space along similar vascular territories as PVFs. Astrocytes (gray) reside in the parenchyma and extend processes to create endfeet that surround the brain vasculature. (**B**) Graphic depicting a cross-section of an arteriole in the upper and deeper cortical layers as well as a venule showing the layering of the vasculature. Endothelial cells (light pink), smooth muscle cells and PVFs are encased in separate BMs (pink and light purple). Astrocytic endfeet deposit extracellular matrix and adhere to the vascular wall. CSF also fills within the PVS. The PVS is denoted in each cross-section with zoomed-in insets for arterioles and venules to depict the basement membrane layers. (**C**) Graphic depicting the pathological changes to the PVS in neurodegenerative diseases. PVFs may undergo some degeneration and aberrant signaling. Degeneration of smooth muscle cells, thickening of the vascular basement membranes, and deposition of proteins in the basement membranes are known to occur. This hinders vasomotion and movement of CSF. Together these likely contribute to enlargement of the PVS which is associated with poor waste clearance. It is unknown if degeneration or aberrant signaling in PVFs plays a role in smooth muscle cell degeneration and/or thickening of the vascular basement membranes.

As for their organization along the brain vasculature, PVFs surround pial arteries extending all along arterioles to their termination points deep into the brain (Figure 1A) (3). Their coverage continues on vessels that branch from penetrating arterioles, known as the arteriole-capillary transition (ACT) zone, and ends prior to the capillary bed, when levels of alpha-smooth muscle actin drop from high to low/undetectable levels (12). PVFs are also found on large diameter venules (≥12 μm), most abundantly on the largest ascending venules, principal cortical venules, that extend from the pial surface down into the underlying white matter in the cerebral cortex (3, 13). Along all vascular zones, PVFs maintain a consistent morphology, with flattened somata and lamellar sheaths surrounding the vessel wall. However, their function along these distinct vascular territories in the healthy brain is largely unknown.

## Perivascular fibroblast dynamics and cellular interactions in the perivascular space

It is well established that PVFs occupy the PVS which is an ECM/CSF-filled space surrounding arterioles and venules continuous with the subarachnoid space (2, 9). The CSF-filled PVS is very apparent in the upper layers of the cortex and eventually decreases in size in the deeper layers becoming mostly basement membrane (Figures 1A,B). In addition to harboring immune cells like perivascular macrophages (PVMs), the PVS is known to participate in brain waste clearance which relies on continual movement of CSF via arteriole vasomotor activity and heartbeat (14–17). PVFs are embedded in the outer basement membrane in between the mural cell layer and the astrocytic endfeet (9) (Figure 1B). Within this space, PVFs exhibit some limited mobility within their territories in the normal brain, which is in contrast to pericytes that appear firmly embedded in their positions (3). This lends to the possibility that PVFs are sensing the perivascular environment, modulating the outer vascular wall, and interacting with other perivascular populations like PVMs, astrocytes, and mural cells.

PVFs and PVMs both reside within the PVS (Figures 1A,B). PVMs are a subset of parenchymal border macrophages and are distinguished by expression of CD206 and Lyve1 (18). They are mainly known for their immune surveillance and phagocytic activity (19, 20). Their phagocytic activity is particularly important for blood-brain barrier (BBB) function in brain regions where vessels lack barrier properties like the area postrema in which PVMs sequester molecules larger than 10kDa (21). PVMs on arterioles also take up fluorescent tracers injected into the caudate putamen, demonstrating a potential role in brain waste clearance (22). Further, depletion of PVMs, in addition to parenchymal border macrophages, resulted in increased expression of ECM genes in PVFs (18). Along with the loss of PVM-derived matrix metalloproteinase proteins, this is thought to stiffen the vascular wall reducing vasomotor activity of arteries and arterioles and thus impairing CSF flow and waste clearance. In non-CNS tissues, studies have revealed that reciprocal signaling between macrophages and fibroblasts is involved in maintenance of cellular quiescence and fibrotic responses during disease (23). Thus, it is likely that PVMs and PVFs regulate the cellular state of one another to maintain homeostasis and function of the PVS. Notably, the spatiotemporal development of PVFs and PVMs along penetrating vessels coincide almost identically (8). Further, timing of PVF and PVM arrival around postnatal day 7 in the brain corresponds to when the PVS begins to clear brain waste (24). Together this suggests PVFs and PVMs respond to similar developmental cues and perhaps establish interactions that are important for the creation, function, and/or homeostasis of the PVS.

Astrocytes ensheath the outermost layer of the vasculature with their endfeet encasing the PVS and bridging the vasculature with neurons (25). Astrocytes, along with PVFs, likely participate in creating the outer basement membrane by depositing laminin (26) (Figure 1B). Further, the majority of astrocytes proliferate and differentiate in the postnatal mouse brain where they begin to assist in neurotransmitter recycling, modulation of synaptogenesis and synaptic transmission, water transport, and BBB maintenance (27, 28). Astrocytes are also well known for regulating blood flow by sensing neuronal activity and subsequent release of vasomodulators on arterioles to increase local blood flow (29). This is called neurovascular coupling and is dependent on gap junctions and hemichannels formed by Cx43, Cx30, and Cx26 proteins to relay electrical signals (K+, Ca2+) throughout the astrocytic networks and expose the vasculature to vasomodulators (30). Based on single cell transcriptomic data, PVFs as well as meningeal fibroblasts highly express Cx43, Cx30, and Cx26 (31). Thus, PVFs may relay vasomodulators and electrical signals through direct communication with astrocytes and smooth muscle cells via gap junctions and hemichannels, respectively. However, it has not been established if direct communication exists between astrocytes, PVFs, and smooth muscle cells, and whether PVFs are involved in blood flow regulation.

PVFs surround the mural cell layer, specifically smooth muscle cells on arterioles, ensheathing pericytes on the ACT zone, and venule stellate mural cells on venules (3) (Figure 1A). Due to their perivascular location and common expression of Pdgfrβ, pericytes and PVFs have been confused with one another, where PVFs are sometimes referred to as “type A pericytes” (32, 33). However, use of Col1a1 and other fibroblast markers like Pdgfrɑ, in addition to their perimural location have allowed for better distinction between the two perivascular cell types (1–3). Mural cells along arterioles and the ACT zone are capable of rapid modulation of blood flow into the capillary network in response to neuronal activity (12). While it is unknown if PVFs actively regulate blood flow in the brain as discussed above, it is possible they play a part by modulating the tension of the arteriole wall. PVFs, mural cells, and endothelial cells are encased within unique basement membranes (34) (Figure 1B). In general, each basement membrane consists of glycoproteins from four major families: laminins, non-fibrillar collagens, nidogens and heparan sulfate proteoglycans. The innermost basement membrane around the endothelial layer predominantly consists of non-fibrillar collagen type IV with laminin α4 and α5. The mural basement membrane is of similar composition but also includes laminin α1 on arterioles (2, 35). Unlike capillaries where the endothelial-pericyte basement membranes are continuous, basement membranes on arterioles ensheath each cell layer and are interconnected to one another through an interstitial matrix made up of fibrillar collagen type I and III (35). The elastin layer is sandwiched between smooth muscle cells and endothelial cells on arterioles, and importantly provides the flexibility necessary for the dynamic diameter changes (36, 37). PVFs are embedded in the outer basement membrane adjacent to the astrocytic endfeet basement membrane layer (9). Through focal adhesions with their underlying ECM, fibroblasts can modulate tension of the basement membrane in response to stimuli (38, 39). Thus, it is possible that PVFs create tone within the outer basement membrane that confines the contractility and dilatory actions of mural cells when modulating blood flow. However, this possibility has yet to be tested.

## Progenitor role for perivascular fibroblasts

On non-CNS vessels in zebrafish, PVFs arise at earlier stages than pericytes and act as progenitors giving rise to pericyte populations (40). The CNS vasculature in zebrafish develops similar BBB properties as mammals with endothelial tight junctions, pericytes, and radial glial contacts that resemble astrocytes (41). To date, PVF coverage on CNS vessels in zebrafish has not been described. Although given that zebrafish and mammals share many of the same brain vascular properties, it is possible PVFs also reside on CNS vessels in zebrafish and give rise to brain pericytes in a similar manner. However, in contrast to zebrafish, smooth muscle cell and pericyte populations are initially established in the brain during embryonic stages in mice, prior to the postnatal arrival of PVFs (8, 42). This does not completely rule out the possibility that PVFs can modulate mural cell populations in later developmental stages, or diseases where mural cell degeneration occurs. Lineage tracing approaches to test this possibility in mammalian models have not been performed to our knowledge.

## Contribution of perivascular fibroblasts to the vascular wall

The outer basement membrane and interstitial matrix of arteries, arterioles, venules, and veins where PVFs are embedded, is critical for providing integrity, facilitating the integration of multiple perivascular cells, and allowing for mechano-physical control of vessel dynamics (9, 34). Due to high expression of many ECM genes in brain fibroblasts, it is hypothesized that they contribute to production and maintenance of this basement membrane and the interstitial matrix (2, 31). These include fibrillar collagens (*Col1a1*, *Col1a2*, *Col3a1*, *Col5a1*, *Col5a2*, and *Col5a3*) and non-fibrillar collagens (*Col6a1*, *Col6a2*, *Col6a3*, *Col8a1*, *Col8a2*, *Col11a1*, *Col12a1*, *Col13a1*, *Col15a1*, *Col16a1*, *Col23a1*, *Col26a1*). Brain fibroblasts also express the collagen-modifying enzymes (i.e., lysyl oxidases) and organizers such as lumican (*Lum*) and decorin (*Dcn*). Recent work has demonstrated that PVFs can be further differentiated from pial fibroblasts for their enriched expression of *Col15a1*, *Col12a1*, *Col4a1*, *Col14a2*, and *Spp1* (31). To our knowledge, only Col1a1 and laminin-ɑ1 are exclusively around arterioles and venules in the brain, but it is unknown if these ECM proteins are solely PVF-derived (2, 31, 43). In zebrafish, PVFs deposit Col1a2 along the vasculature and loss of PVFs results in dysmorphic vessels (40). This demonstrates that PVFs are likely crucial for the vascular integrity of dynamic vessels by supplying ECM proteins like Col1a2 (44).

## Profibrotic role for perivascular fibroblasts in the central nervous system

PVFs gained attention for their profibrotic response following spinal cord injury in studies by Soderblom et al. (1). In these studies, spinal cord injury was induced without puncturing the surrounding fibroblast-rich meninges resulting in a proliferative and fibrotic response by PVFs, which secreted collagen I in place of the damaged, dying tissue, ultimately creating the fibrotic scar. Participation of PVFs along with meningeal fibroblasts in creating the fibrotic scar is now known to be a common phenomenon following stroke, neuroinflammation, and intracerebral hemorrhage (43, 45–48). Interestingly, fibroblasts assist in BBB repair following stroke and intracerebral hemorrhage (48, 49). However, the fibrotic scar is not permissive to axonal regeneration and remyelination following spinal cord injury (46, 47, 50). Blocking the proliferation of profibrotic cells improved axonal sensorimotor recovery. However, this also prevented complete sealing of spinal cord lesions (32). Together, these studies suggest that fibroblasts, including PVFs, may have some protective roles for brain vascular repair but their fibrotic activity likely prevents neuroregeneration.

A perivascular origin for pro-fibrotic, collagen producing cells has also been described following injury of peripheral organs such as the skin, skeletal muscle, heart, kidney, liver, and lung (51). Similar to the brain, PVFs in these peripheral tissues also reside on large diameter vessels like arteries, arterioles, venules and veins and express Pdgfrα under homeostatic conditions. In the adventitia of large peripheral arteries, fibroblasts deposit collagen among other ECM proteins to maintain the structural integrity and functionality of these large vessels (52). However, fibroblasts in the periphery and brain, are quite heterogenous and thus a shared marker for PVFs among all the organs has yet to be identified (31, 53). Identifying better markers will help us understand the differences and commonalities of PVFs in the CNS and periphery in health, injury, and disease.

## Thickening of the vascular basement membrane in AD

Thickening of the vascular basement membranes is commonly observed along the vasculature in Alzheimer's Disease (AD) (34, 54) (Figure 1C). Studies have reported increased deposition of collagen IV, fibronectin, and the heparan sulfate proteoglycans, agrin and perlecan (55–60). Other than collagen I, which was increased in proteomic studies on the brain microvasculature from AD patients, very little is known about PVF-specific ECM gene changes (34, 54). Nonetheless, it is hypothesized that disorganization, thickening, and altered deposition of ECM components affects brain waste clearance and leads to excessive vascular accumulation of amyloid-β (Aβ), particularly within the ECM of arteries and arterioles (61–63). Build-up of Aβ along the brain vasculature is a major characteristic of cerebral amyloid angiopathy (CAA) and is associated with smooth muscle cell degeneration and poor vasomotor activity (64) (Figure 1C). The fate of PVFs in AD and CAA, and whether they play a part in these pathological events has not been well explored. Single cell transcriptomic studies have shown that PVF populations are reduced in postmortem AD tissue, along with a reduction in endothelial and mural cells, with the remaining PVF populations exhibiting heightened interferon and SMAD signaling (65). It is possible that the PVF layer undergoes some degeneration, and that aberrant signaling in the remaining population results in thickening of the basement membrane. This could affect the compliance of the vascular wall. Thus, understanding the role and timing of PVF influence in AD and CAA pathology could be of importance in devising ways to improve vascular function in patients with AD.

## A role for perivascular fibroblasts in the enlargement of the perivascular space in disease

Enlargement of the PVS has been associated with a range of nervous system diseases and cognitive decline (66, 67). Notably, enlargement of the PVS and activation of PVFs was recently observed in mouse models and postmortem tissue from patients afflicted with Amyotrophic Lateral Sclerosis (ALS) (68). In these studies, PVFs increased expression of Col6a1 and Spp1, a protein involved in bone mineralization, prior to ALS symptom onset. This suggests that PVFs become activated early in ALS, and biomarkers such as Spp1 could be an indicator of ALS progression. Enlargement of the PVS is also a feature of cerebral small vessel disease (CSVD) which, in addition to other vascular pathologies, is implicated in both vascular dementia and AD (66). CSVD, which includes CAA, is associated with vascular stiffening, inflammation, protein deposits (such as Aβ), loss of mural cells, and disruption of the BBB. Collectively, these features are hypothesized to drive PVS enlargement due to poor vasomotor oscillations and stalling of CSF flow, thus creating a vicious cycle that hinders PVS waste clearance and neurovascular regulation (67, 69) (Figure 1C). In particular, PVS enlargement is regularly observed along cortical arterioles in white matter regions, correlating with vascular Aβ deposition and reduced smooth muscle cell coverage (70). PVS enlargement was also associated with heightened tau and Aβ pathology as well as cognitive decline, suggesting enlargement of the PVS exacerbates AD progression (71–73). It is plausible that aberrant signaling or degeneration of PVFs in neurodegenerative diseases is involved in the enlargement of the PVS through various mechanisms in AD, such as smooth muscle cell degeneration and/or thickening of the vascular ECM ultimately attenuating vasomotion and CSF movement. However, more thorough studies are needed to understand the relationship between PVFs and PVS enlargement.

## Conclusion

We have discussed the characteristics and potential roles of PVFs in supporting the brain vasculature. We have highlighted how PVFs are likely derived from the pia during postnatal development, and along with PVMs, may play a role in creating and maintaining the functionality of the PVS. We noted a potential role for PVFs in regulation of cerebral blood flow through signaling interactions with astrocytic endfeet and SMCs, in addition to regulating the tone of the arteriole wall. Further, the high expression of various ECM proteins and regulators in PVFs points to their probable role in maintaining the integrity of the vascular wall. Thus, abnormal function or density of PVFs may be involved in thickening of the vascular basement membranes and enlargement of PVSs that is observed in AD, CAA, and other neurodegenerative diseases. Future studies aimed at uncovering the role of PVFs in health and disease will be instrumental for understanding how these elusive cells contribute to vascular homeostasis and pathology.
